# Recent advances in nanogels in veterinary medicine

**DOI:** 10.1186/s13567-025-01576-y

**Published:** 2025-08-07

**Authors:** Maria Laura Soriano Pérez, Maria Carolina Flores Bracamonte, Romina Bellingeri, Fabrisio Alustiza, Maria Molina

**Affiliations:** 1 Grupo Sanidad Animal, INTA Marcos Juárez, Córdoba, Argentina; 2 Instituto de Investigaciones en Tecnologías Energéticas y Materiales Avanzados (IITEMA), Universidad Nacional de Río Cuarto (UNRC) - Consejo Nacional de Investigaciones Científicas y Técnicas (CONICET), Río Cuarto, Córdoba Argentina

**Keywords:** Nanogels, vaccines, mastitis, One Health, mucosal administration

## Abstract

Nanogels, a promising class of nanomaterials, have emerged as versatile platforms in veterinary medicine and have shown substantial progress in recent years. This review outlines the key developments and potential applications of nanogels in veterinary therapeutics. It provides an in-depth discussion of critical factors influencing nanogel implementation, including synthesis, biocompatibility, biodegradability, clinical translation, and technological considerations. Approaches to nanogel-based vaccines and drug-delivery systems are examined, with particular emphasis on the mucosal route of administration, given its relevance in livestock. Finally, the integration of nanogels into the One Health framework is considered.

## Introduction

**Figure 1:**
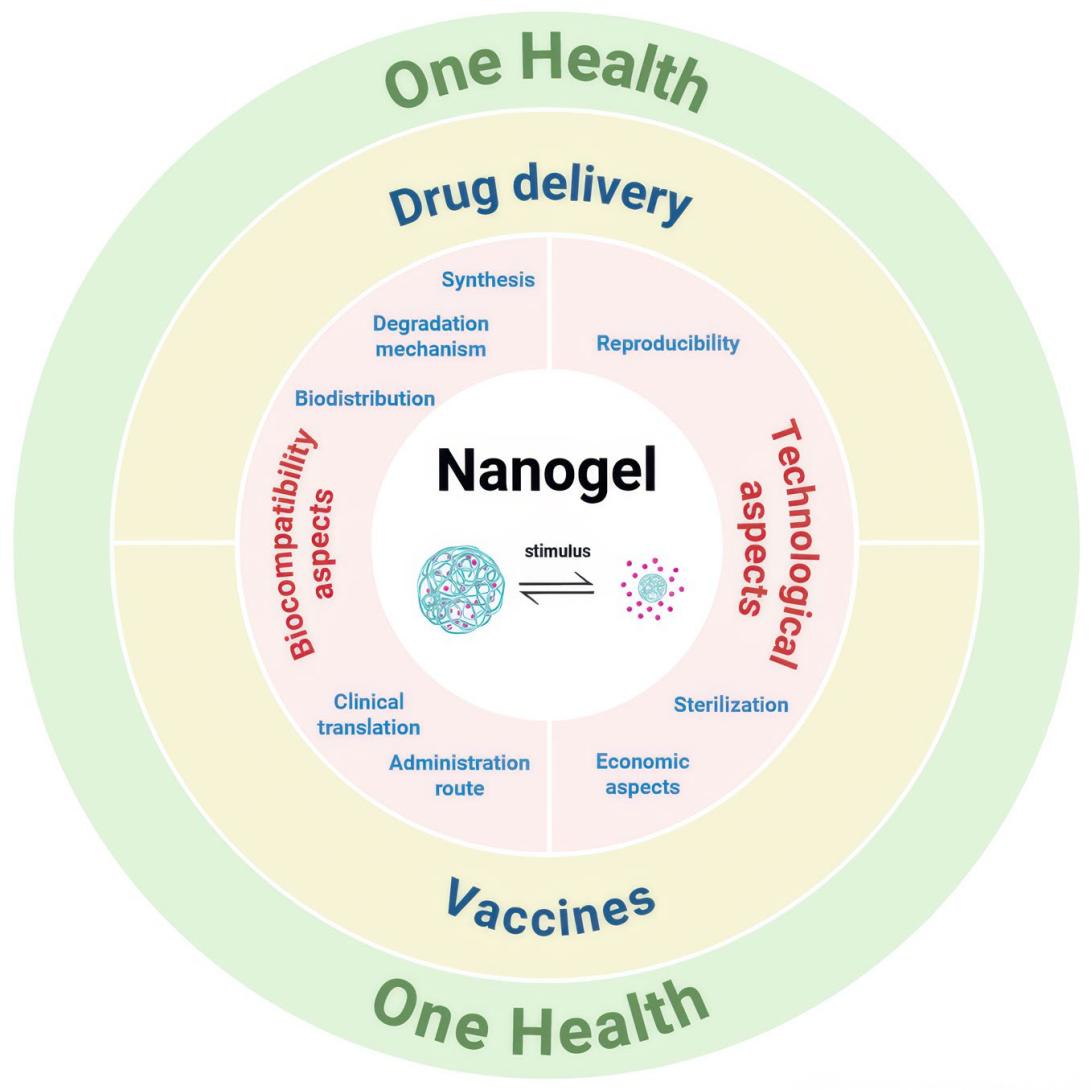
**Nanogels and their applications in veterinary medicine from a ‘'One Health'’ perspective.**

The convergence of nanotechnology and veterinary medicine has precipitated a paradigm shift in developing advanced therapeutic strategies. Among various innovative approaches, nanogels have emerged as a versatile and promising platform with substantial potential to transform veterinary healthcare. This review provides a comprehensive exploration of the multifaceted applications of nanogels in veterinary medicine (Figure 1).

The field of nanotechnology has expanded rapidly due to its ability to engineer materials at the nanoscale, conferring unique physicochemical properties that enhance biocompatibility, stability, and targeted functionality. Nanogels, three-dimensional networks of polymers or biopolymers, represent a distinctive class of nanomaterials that have garnered considerable attention for their inherent ability to encapsulate, protect, and deliver therapeutic agents with unparalleled precision [1]. In veterinary medicine, nanogels offer promising solutions to a range of challenges, from adjuvant delivery [2, 3] to controlled drug release [4]. Moreover, nanogels can be engineered to respond to external stimuli such as pH, temperature, or enzymatic activity, enabling on-demand drug release at specific target sites [5].

In recent years, numerous studies have focused on the synthesis, characterisation, and biological applications of nanogels for human health. However, to the best of our knowledge, no consolidated review has been published on the use of nanogels in veterinary medicine.

Accordingly, this review highlights the diverse strategies employed in the design and application of nanogels for veterinary use. It first presents an in-depth discussion of the key factors in nanogel implementation, including synthesis, biocompatibility, biodegradability, clinical translation, and technological considerations. Approaches to nanogel-based vaccines and nanogels as drug-delivery systems are then examined, with emphasis on the mucosal route of administration, given its relevance in animals. Finally, the potential role of nanogels within the One Health framework is discussed.

## Key aspects in the implementation of nanogels in veterinary medicine

For the implementation of nanogels in veterinary medicine, it is essential to consider key aspects such as biocompatibility and technological factors. Designing biocompatible nanogels involves considering the synthesis mechanisms, biodistribution, degradation mechanisms, administration routes, and clinical translation. Furthermore, it is crucial that biocompatible nanogels are reproducible and maintain their integrity when exposed to different sterilisation methods. Finally, economic factors play an essential role in ensuring that nanogels are cost-effective and accessible in veterinary practice.

### Biocompatibility aspects

Nanogels have emerged as a versatile hydrophilic platform with promising biomedical applications. Despite extensive research into their therapeutic potential, several challenges remain in developing an optimal delivery system. The interactions between nanogels and their biological environment depend on a complex interplay between the controllable properties of the particles and the largely uncontrollable characteristics of the surrounding biological media.

#### Synthesis of nanogel

Nanogels are generally highly hydrophilic; in a biological environment, they swell and retain large amounts of water, contributing to their biocompatibility [6]. However, nanogel synthesis often involves monomers, initiators, and cross-linkers that may exhibit toxic characteristics [7]. To ensure safety and efficacy, the polymers employed in nanogel synthesis must be both biocompatible and biodegradable. Biopolymer-based nanogels offer several advantages, including inherent biodegradability, abundance in nature, renewability, non-toxicity, and relative cost-effectiveness. Naturally derived nanogels can be prepared from protein and polymers such as collagen, albumin, fibrin, chitosan, hyaluronic acid, heparin, chondroitin sulfate, agarose, and alginate [8].

Dextran, a saccharide widely used in drug development, is approved for medical use by the Food and Drug Administration (FDA). Dextran-based nanogels have demonstrated broad biomedical applications, including cell encapsulation, tissue engineering, and drug delivery, and have shown good biosafety profiles following intravenous (IV) administration [9]. Compared with natural polymers, synthetic polymers offer lower or more controllable immunogenicity, as well as tunable composition and degradability [10]. As a result, there is growing interest in the development of biodegradable nanogels based on synthetic polymers for biomedical applications.

Cross-linkers play a crucial role in stabilising binding sites and controlling the structure of the polymer matrix within nanogels. By tuning the internal cross-linking within the nanogel network, drug release can be regulated in response to specific stimuli. Chemically cross-linked nanogels formed via covalent bonds exhibit colloidal stability under in vivo conditions, preventing rapid dissociation of the gel network. Studies on mouse models have shown a direct correlation between higher cross-linker density and prolonged circulation half-life in hybrid nanogels carrying gold nanoparticles [11]. These findings suggest that optimising cross-linker density can enhance the stability and efficacy of nanogel-based drug-delivery systems.

A key consideration in nanogel manufacturing is ensuring that the final product is free from residual solvents or unreacted monomers, as these can impart toxicity [12]. Various synthesis methods have been reported for the preparation of polymeric nanogels, including emulsion polymerisation, precipitation polymerisation, self-assembly, and one-step polymerisation in homogeneous solution. Among these, the emulsion polymerisation method may result in unreacted monomers and oligomers that could pose toxicity risks in clinical applications [13]. Leading strategies are discussed to provide new solutions for critical healthcare scenarios, aligned with final curative aims while maintaining the essential criteria of biocompatibility and biodegradability [14].

#### Biodistribution and circulation time

Nanogels can prolong circulation half-life by preventing rapid clearance, degradation, or metabolism. One of the most significant obstacles to achieving sustained circulation is the process of opsonisation, which facilitates phagocytosis and binding with serum proteins. This, in turn, promotes uptake by cells of the mononuclear phagocyte system (MPS), including monocytes, macrophages, and dendritic cells [15].

The surface charge of nanogels plays a critical role in modulating their opsonisation profile, thereby influencing recognition by MPS cells and altering their plasma circulation profile. Gel particles with a neutral surface charge have been shown to exhibit longer circulation times. PEGylation (polyethylene glycol modification) of the nanogel surface enhances hydrophilicity, shields any core charge, and establishes steric hindrance that reduces interaction with serum proteins [16]. As central cellular mediators of host exposure, phagocytes influence both biodistribution and the balance between host tolerance and potential nanotoxicity. Macrophages, as primary and initial cell types that interact with nanoparticles, are key regulators of the host’s inflammatory and immunological responses to nanogels [17].

Beyond surface charge, additional parameters such as particle size and stability determine the biodistribution of nanogels following administration [18]. The softness and deformability of nanogels directly influence their biodistribution and circulation time within the body. These properties help nanogels partially evade the splenic filtration process. Hendrickson and Lyon [19] demonstrated that nanogels subjected to forces close to the renal filtration pressure could pass through pores over 10 times smaller than their size. In vivo studies further indicate that softer nanogels pass through physiological barriers, particularly splenic filtration, more easily than stiffer counterparts due to their deformability, resulting in extended circulation half-life and reduced splenic accumulation. Moreover, the smaller tunable size of nanogels results in increased blood circulation time post-administration, with the potential for active or passive targeting to specific sites of action [20]. This outcome results in better cellular uptake and reduced elimination by the reticuloendothelial system [21].

The elasticity of nanogel is another determinant of biodistribution. Anselmo et al. [22] developed PEG-based hydrogel nanoparticles with variable elastic moduli by altering the volume fraction of poly(ethylene glycol) diacrylate, demonstrating that softer particles exhibited prolonged circulation compared to hard nanogels of equivalent size.

**Figure 2:**
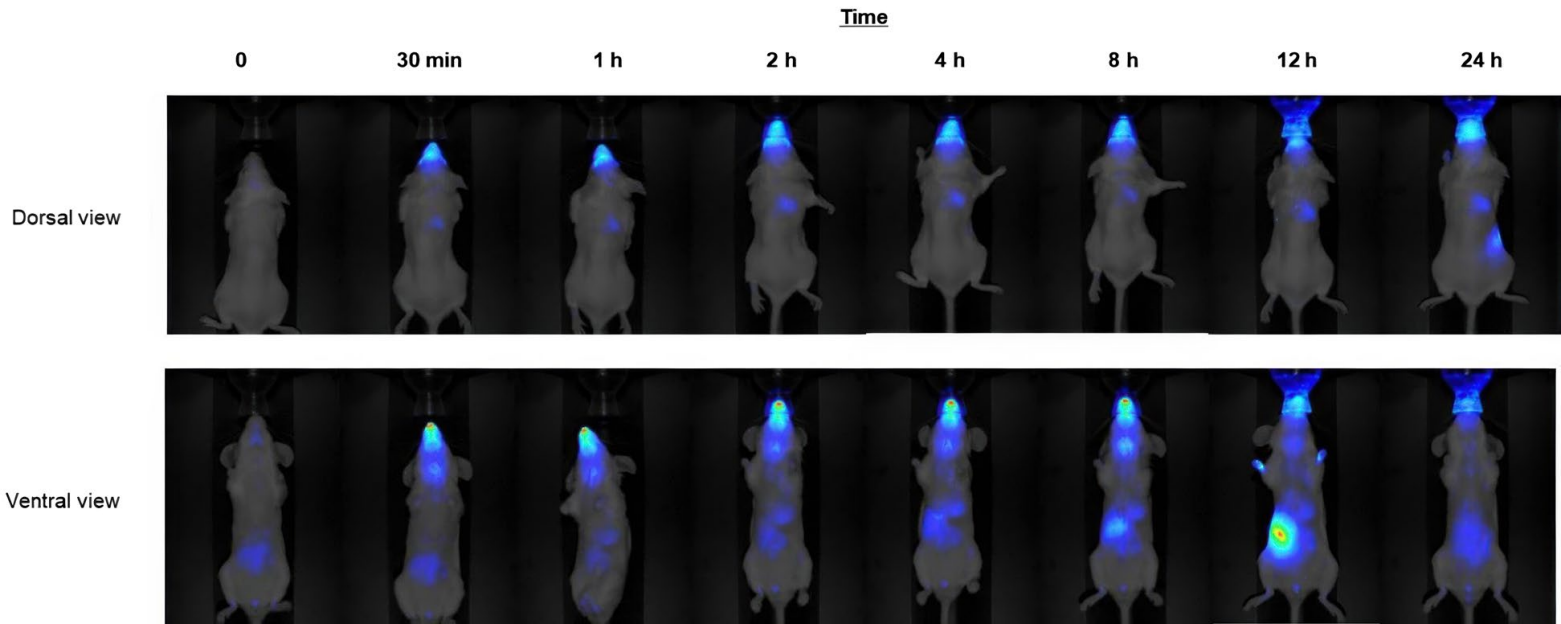
**Intranasal administration of pNIPAM nanogels**. Images acquired at different time points from dorsal and ventral views in pseudocolour of mice. Reprinted with permission from reference [2].

Within our research group, Soriano et al*.* [2] developed thermosensitive nanogels based on poly (N-isopropylacrylamide) (pNIPAM) for use as vaccine nanocarriers targeting the outer membrane lipoprotein A (OmlA). This protein is a key virulence factor of *Actinobacillus pleuropneumoniae* (App), the causative agent of porcine pleuropneumonia. In the study, the biodistribution of these nanogels was evaluated in female BALB/cCmedc mice following intranasal administration. In vivo imaging was performed at various time points post-application. Signals were observed in the lungs within an hour of administration and remained elevated for up to 24 h. By 12 h, a stronger signal was detected in the intestinal region and faeces, suggesting elimination of the nanogels via the digestive system (Figure 2).

#### Degradation mechanism

The design of nanogels must consider the ideal clearance mechanism, which depends on the intended biomedical application. For example, in diagnostic and imaging applications, when only first-pass distribution is required, small, actively targeted particles that can be rapidly cleared are preferable. Conversely, prolonged circulation times are generally desired for drug-delivery vehicles to enable greater accumulation at the disease site.

The degradability of nanogels is an important factor in minimising the toxicities associated with their accumulation in animals. To ensure effective elimination and high tolerance, nanogels must be synthesised using materials that are biocompatible and biodegradable. The degradation behaviour of polymeric networks depends on the specific polymers and cross-linking agents used. The network structure can be disintegrated by incorporating labile cross-linking points into the nanogel structure, yielding freely soluble polymer chains [23].

The molecular weight of nanogels typically exceeds the renal threshold (∼40 kDa for copolymers), preventing their removal through the kidneys. If the polymers are non-degradable, they tend to accumulate in the animal tissues and organs. Even when nanogels are engineered to degrade into smaller polymer fragments, there remains a risk of cellular accumulation of polymer chains due to sequestration in lysosomal compartments. For this reason, the use of biodegradable polymers, both natural and synthetic, is preferable. It is important to note, however, that chemical functionalisation could alter the rate and pattern of polymer degradation, potentially rendering a natural polymer non-biodegradable [24]. Consequently, a detailed evaluation of the metabolic fate and elimination profile of nanogels is essential before nanogels can be recommended for long-term clinical use.

#### Administration route

Nanogels are promising candidates for the controlled delivery of active compounds such as drugs, proteins, and nucleic acids due to their favourable physicochemical properties and biocompatibility. Nanogels loaded with active agents can be administered through various routes, including mucosal, transdermal, and intravenous pathways. This section focuses on mucosal administration, which, despite its advantages, remains relatively underexplored in veterinary medicine.

Mucosal drug delivery is a valuable strategy for targeting treatments to specific areas like the gastrointestinal tract, reproductive system, respiratory system, bladder, and eyes. The mucus barrier covering these surfaces is a complex viscoelastic gel composed of water, proteins, carbohydrates, salts, antibodies, bacteria, and cellular debris. It consists of two layers. The first, the sol layer, lies in direct contact with the epithelium, has a low exchange rate, and is more fluid, facilitating ciliary movement. The second, the external (gel) layer, is thicker and formed by salt, antibodies, and mucin. Its principal function is to prevent bacterial entrance [25]. Mucins, negatively charged glycoproteins, comprise approximately 80% carbohydrates and 20% of a linear protein core [26].

Cuggino et al. [27] extensively reviewed mechanisms aimed at achieving primarily two properties of nanogels: mucoadhesion and mucopenetration.

Mucoadhesion is a complex mechanism involving the adhesion between mucus and nanogel through covalent or non-covalent interactions such as Van der Waals forces, hydrogen bonding, and hydrophobic interaction. To enhance this bioadhesion, mucoadhesive materials are used to increase contact between nanoparticles and mucosal tissue. Among polymeric materials, positively charged polymers such as chitosan have been widely studied for their potential adhesiveness due to the ability of their amino groups to establish ionic interactions with sialic and sulfonic acids in mucin glycoproteins. In addition to its mucoadhesive potential, chitosan offers good biodegradability and biocompatibility [26]. Negatively charged polymers such as sodium alginate, carboxymethylcellulose, and poly(acrylic acid) are also employed due to the presence of carboxylic acid side groups. Sodium alginate, for example, possesses adhesive properties alongside good biocompatibility [26, 28].

Mucopenetration mechanisms comprise passive and active processes. Passive methods aim to reduce interactions between the nanogel and the mucus or epithelium, facilitating their penetration. Strategies, such as coating with polyethylene glycol (PEG), are employed, which gives the nanoparticles a hydrophilic surface and a neutral charge, avoiding hydrophobic and electrostatic interactions with the mucus [29]. Another strategy involves designing virus-mimicking nanogels with a neutral zeta potential and functionalised surfaces [27]. However, such approaches have seen limited application in veterinary contexts.

In contrast, active mucopenetration relies on interactions between nanogels and the mucus or epithelium that induce chemical changes. One approach involves using mucolytic enzymes bound to the surface of nanoparticles, which are capable of degrading mucin and facilitating deeper penetration. For instance, Charbaji et al*.* [30] developed poly(N-isopropylmethacrylamide) (PNIPMAM) nanogels based on dendritic polyglycerol (dPG) with disulfide groups (PNIPMAM-(SS)-dPG), incorporating disulfide as permeation enhancers. These nanogels demonstrated high biocompatibility and efficient penetration in freshly excised porcine small intestine gastrointestinal mucus models and reconstructed human bronchial epithelium models, whereas control nanogels lacking disulfide did not. It is suggested that disulfide bonds favour interaction with mucin and, being susceptible to mucosal glutathione, facilitate nanogel degradation and controlled release of charged molecules.

### Technological aspects

Despite a growing number of scientific papers describing new formulations, synthetic methodologies, and potential applications of nanogels, only a limited number have progressed to clinical trials [31–35]. Significant challenges remain, including the need for robust clinical data and the resolution of critical issues related to pharmacodynamics, metabolism, and pharmacokinetics. Addressing these limitations is essential before nanogels can advance from clinical trials to widespread routine clinical applications in veterinary medicine. The reproducible and consistent manufacturing of nanosystems remains a key hurdle that must be addressed.

Scaling up the production of nano-objects presents considerable technical challenges. As production scales increase, the formation of novel surfaces becomes less favourable relative to bulk phase processes. One promising strategy that ensures controllable and large-scale production of nanogels involves using microfluidic techniques. Importantly, microfluidic systems also enable scalable, reproducible production of nanogels, facilitating the transition from laboratory-scale synthesis to industrial manufacturing of hydrogel-based nanocarriers [36, 37].

### Economic aspects

Nanotechnology offers transformative solutions to longstanding challenges in veterinary medicine. However, several economic barriers must be overcome to ensure the success of nanomedicine in mainstream veterinary practice. At every stage of drug discovery and commercialisation, it is crucial to secure intellectual property rights through timely patent filing. This step is critical given the protracted timeline required to achieve regulatory approval, complete necessary clinical trials, and introduce a new pharmaceutical to the market [38].

For the market to grow further, risk issues should be addressed, and the broader social implications should be evaluated. Given the high prices of nanomaterials and nanomaterial intermediates, a thorough analysis of the cost-effectiveness and the required purchasing power of animal healthcare systems is essential [39]. Collaborative efforts between the medical research community and the veterinary pharmaceutical industry are urgently needed to avoid missing opportunities for successful future innovations in nanomedicine.

## Application in veterinary medicine

In recent years, a variety of nanogels have been studied for different applications in the field of biomedicine, with increasing interest in their use within veterinary medicine. These include roles as vaccine platforms, adjuvants, drug-delivery systems, and more. Reported studies encompass both biocompatibility assays in different cell lines and in vivo assays in animal models, including mice, cows, and equines (Table 1).

**Table 1:** **Summary of nanogels application in veterinary medicine**.

Application	Nanogel composition	Stage of development	Cell line / Animal model	Key findings	Reference
Intranasal or intraperitoneal vaccine against *Neospora caninum* tachyzoites as a possible treatment for infections in production animals	Chitosan-based nanogels decorated with alginate or alginate-mannose	In vivo	BALB/c mice	Vaccination with recNcPDI using nanogels showed a notable beneficial effect, reducing clinical signs and parasite load in the brain	[52]
Intranasal vaccine against porcine pleuropneumonia	Nanogels based on poly(N-isopropylacrylamide) (pNIPAM)	In vivo	BALB/c mice	Biocompatibility and effective immune response	[2]
Intranasal vaccine against porcine pleuropneumonia	Nanogels based on poly(N-isopropylacrylamide) (pNIPAM)	In vivo	Equine and murine	Nanogels are effective carriers for antigen-based vaccines, are biocompatible, possess high affinity for antigen-presenting cells, and do not induce inflammation at the injection site	[3]
Nanogels as delivery system for enrofloxacin to improve antibiotic efficiency against mastitis cows caused by intracellular *S. aureus* SCVs	Gelatin-SA composite nanogels	In vitro	RAW 264.7 macrophages cell line	Nanogels loaded with enrofloxacin are internalised more efficiently into cells and could enhance the antibiotic’s bioadhesive capacity to SASCVs	[63]
Nanogels as delivery system for tilmicosin against *Staphylococcus aureus* cow mastitis	Self-Assembly Sodium Alginate-Chitosan Nanogel	In vivo	China Holstein cows	Research suggests TIL nanogel could reduce the dose of tilmicosin for bovine mastitis caused by *S. aureus*	[64]

Among the recent scientific developments, several have progressed to patented technologies. In this context, innovative polymeric platforms for pharmaceutical and veterinary applications have been designed. One such technology, detailed in a recent patent [40], involves nanometric copolymer networks comprising at least one segment of a polyamine polymer and another of a non-ionic, water-soluble polymer. These networks do not introduce new pharmacologically active compounds but rather improve the performance of existing drugs by enhancing properties such as solubility, bioavailability, enzymatic stability, toxicity, and site-specific delivery. By incorporating a bioactive molecule, these systems allow for the development of compositions with optimised therapeutic profiles. In addition, they can encapsulate a wide range of substances—from small molecules to proteins, polysaccharides, and nucleic acids—making them highly versatile and applicable across biomedical and veterinary fields.

Another patented invention [41] describes the development of hydrogels composed of graphene, chitosan, and polyethylene glycol diacrylate (PEGDA), referred to as PCG Hydrogel, alongside a thermoresponsive variant containing poly(N-isopropylacrylamide) (pNIPAM), known as TPCG Hydrogel. These systems not only promote the differentiation of mesenchymal stem cells into multiple lineages (adipocytes, chondrocytes, osteocytes) but also function as thermally triggered drug-delivery systems. A notable advantage of these hydrogels is their use of pharmaceutically acceptable excipients and carriers, rendering them suitable for both human and veterinary use. This enables the development of safe, non-toxic, and highly functional formulations for the controlled administration of active pharmaceutical ingredients in animal health.

### Nanogel-based vaccines

Vaccination represents one of the most significant advancements in reducing the burden of infectious diseases across populations. Ongoing research continues to focus on developing safer, more efficient vaccines, prioritising this area of study. In veterinary medicine, the lack of effective vaccines for several pathogens is a persistent issue, further exacerbated by the increasing problem of antibiotic resistance. Animal vaccination plays a crucial role not only in improving animal health and welfare but also in protecting public health by reducing the risk of zoonotic disease transmission [42]. Consequently, veterinary medicine is actively working to expand and incorporate new and advanced vaccination technologies to address these needs [43]. The development of efficacious and safe vaccines administered via non-invasive routes is also gaining traction within the animal welfare framework.

In this context, nanogels have emerged as promising candidates for the development of innovative vaccination strategies. Their large surface area, adjustable size, and ability to encapsulate and release various types of antigens enhance the immune response. In addition, their biocompatibility, biodegradability, and potential for targeted delivery improve vaccine efficacy while minimising adverse effects [44].

#### Nanogels as adjuvant

The traditional mechanisms of vaccine production have primarily relied on the use of whole-attenuated or inactivated pathogens. While these vaccines have been effective in many cases, they are also associated with notable disadvantages, including high reactivity, the need for refrigeration, and the risk of reversion to pathogenic forms. To overcome these limitations, the development of subunit vaccines, particularly those based on protein antigens, has emerged as a promising alternative [45]. These vaccines contain selected non-infectious protective antigens that induce a strong immune response without the risk of disease reversion and with lower storage requirements. However, their low immunogenicity poses a significant challenge. Therefore, adjuvants are necessary to enhance the immunogenicity of these vaccines.

Traditionally, adjuvants have been used to increase the immune response to vaccines with minimal doses and interventions. Furthermore, one of the main objectives of the adjuvants is to direct the type of immune response generated, ensuring it is protective [46]. However, classical adjuvants are known to generate tissue reactions. For example, traditional aluminium salts have been shown to induce necrotic cell death at injection sites [46]. In contrast, results consistently demonstrated the absence of cytotoxic damage and a lack of chronic inflammation when using nanogels [2].

Certain nanogels have demonstrated immunological activity due to their inherent physical–chemical characteristics [44]. Moreover, they exhibit the capability of selectively targeting various cell types and cellular compartments. Their primary advantage lies in their ability to encapsulate and protect protein and vaccine antigens from degradation [47]. The safety profile and efficient uptake by mammalian cells, leading to the induction of adaptive immune responses, are crucial features that position nanogels as strong candidates for vaccine applications. Additionally, they can achieve controlled release triggered by external stimuli, such as temperature or pH, earning them the designation of ‘smart nanogels’ [21]. Through this mechanism, nanogels can control the duration, site, and dose of antigen release, supporting antigen translocation to lymph nodes.

Understanding the interaction between nanogels and the immune system is pivotal to advancing our understanding of nano-vaccination [48]. Serving as a bridge between the innate and adaptive immune systems, further investigation into the interaction between nanogel- and antigen-presenting cells (APC) holds promise for advancing novel and successful nano-vaccination strategies. Nanogels play a significant role in modulating the activity of APC [49]. They elevate the levels of co-stimulatory molecules on these cells, thereby enhancing T-cell activation. Another mechanism through which nanogels exert immune action on APC involves loading, carrying, or decorating with molecules that can induce dendritic cell activation. This process triggers particle-specific immune recognition and antigen processing. These immune stimulatory molecules can be incorporated or encapsulated using various forms of functionalised nanogels [50]. As previously discussed, nanogels are well-suited to protect and transport antigens. Through bioconjugation with antibodies or specific ligands, their active targeting specificity can be further increased [51]. Several studies have demonstrated that nanogels can be functionally modified to elicit diverse responses from the immune system [51].

Durán-Lobato et al. [52] designed an oral vaccine based on poly(2-hydroxyethyl methacrylate-co-methacrylic acid) [P(HEMA-co-MAA)] nanogels. These nanogels were functionalised with mannan on their surface to enhance the uptake by M cells located on the protective surfaces of mucosa-associated lymphoid tissue (MALT). Delivery specificity was further increased by targeting the carbohydrate receptors present on APCs. The surface conjugation of nanogels with mannan, mimicking “pathogen-like” structures, yielded promising results for the development of a safe and effective oral vaccine. Moreover, the use of nanogels augmented macrophage internalisation and up-regulated the expression of co-stimulatory molecules relevant to immune activation.

The use of Toll-like receptor (TLR) agonists is a widely employed strategy in adjuvant development, and their conjugation with nanogels provides an effective means of stimulating the innate immune system [46]. Nuhn et al. [53] developed a nano-vaccine by conjugating an imidazoquinoline-based TLR7/8 agonist to degrade polymeric nanogels of approximately 50 nm in size, which were prepared by self-assembly of amphiphilic block copolymers. Their experiments demonstrated increased T-cell responses and enhanced antibody production against a tuberculosis antigen.

#### Nanogel-based vaccines for diseases

A subunit vaccine for veterinary use against neosporosis was developed by Debache et al. [54]. The vaccine used a recombinant protein antigen in combination with chitosan nanogels as an adjuvant against challenge with *Neospora caninum* infection*. N. caninum* is an obligate intracellular protozoan parasite affecting livestock and companion animals [55]. Neosporosis primarily impacts cattle, the most common intermediate host, and is associated with abortion storms in a naïve herd. Consequently, this parasite represents a significant veterinary concern due to the substantial economic losses caused by reproductive failure in livestock [56].

Although moderately effective live vaccines exist, they present typical challenges associated with whole-attenuated vaccines, including logistical constraints and the risk of reversion to pathogenic forms [54]. To overcome these limitations, recent research on *Neospora* vaccines has focused on recombinant antigens for subunit vaccines. Debache et al. [54] identified the protective function of recombinant Neospora caninum protein disulphide isomerase (recNcPDI), a protein secreted by the parasite that was shown to be involved in interactions between *Neospora* and host cells. They used recNcPDI as a novel model antigen encapsulated in chitosan-based nanogels decorated with alginate or alginate-mannose.

Thirteen groups of BALB/c mice were immunised by two different ways of application: intranasal (i.n.) or intraperitoneal (i.p.) with the acute disease model of cerebral infection in non-pregnant animals. Two types of chitosan nanogels were designed: one surface decorated with alginate and the other surface decorated with mannose-alginate. The vaccination schedule included doses on days 1, 15, and 30, followed by i.p. challenge on day 46 with freshly purified *N. caninum* tachyzoites.

They noted the lack of protection when the antigen alone is applied via i.p., whereas encapsulation of recNcPDI in nanogels increased survival rates. This outcome suggests that the nanogels enhance vaccine efficacy, which could be due to the strong innate immune response generated. Animals vaccinated i.p. with recNcPDI associated with both types of nanogels exhibited reduced clinical signs, and although statistical significance was not reached, the cerebral parasite burden diminished. For i.n. vaccination, the survival rates were high even in cases where a low dose of protein was applied, highlighting a key advantage for instances where the antigen amount is limited. It is important to note that additional adjuvants were used in this study: saponin adjuvant (SAP) for i.p. application and cholera toxin adjuvant (CT) for i.n. delivery. Thus, the profiles of immune responses may be altered or unaffected by the use of nanogels alone. Nonetheless, the authors concluded that the immune response elicited by recNcPDI delivered in nanogels was associated with protection against neosporosis.

As demonstrated in this section, several of the nanogel-based vaccines presented are administered intranasally, the same route by which many pathogens enter the host via mucosal sites. In this context, mucosal vaccination is particularly important as it promotes a protective immune response at the site of pathogen entry, thereby reducing or preventing pathogen invasion and dissemination.

Most current vaccines are administered by injection into non-mucosal sites, which carries certain disadvantages, including antigen degradation that may compromise the vaccine’s protective efficacy. In contrast, nanogel-based vaccines offer several advantages for mucosal immunisation over the parenteral route [57]. When delivered via mucosal sites, nanogel vaccines have the potential to induce systemic and mucosal immunity. Both could be activated and in some cases, even on other distal mucosal surfaces, the response could be measured. Nanogels can preserve protein structure in biological tissues and ensure controlled antigen release, thus initiating a strong immune response. One of their key advantages is their ability to apply through the mucosa due to their resistance in biological environments. Once entering the animal via oral or nasal routes, the cells that respond to this stimulus are included in MALT [58]. MALT is organised into inducer and effector sites; effector sites may include distant mucosal surfaces, which are different from inducer sites through the ‘common mucosal immune system’.

Secretory immunoglobulin A (sIgA) plays a central role in defence against mucosal pathogens. The body secretes IgA to make it more resistant to degradation. It is secreted in a form resistant to enzymatic degradation and functions to inhibit microbial adherence and reduce the activity of microbial enzymes and toxins [59]. Thus, a mucosal vaccine has the potential to induce a strong and effective immune response, both mucosal and systemic, measurable not only by serum-specific levels of IgG but also by the presence of high titers of specific sIgA on surfaces.

The limited availability of nanogel-based vaccines in veterinary medicine presents a significant opportunity for the development and production of new immunogens. These efforts may be directed towards individually tailored applications for companion animals or large-scale deployment in food-producing animals [60]. However, species-specific anatomical differences and practical challenges in administration must be considered when evaluating product applicability. These factors continue to pose obstacles to the widespread adoption of veterinary nano-vaccination strategies.

**Figure 3:**
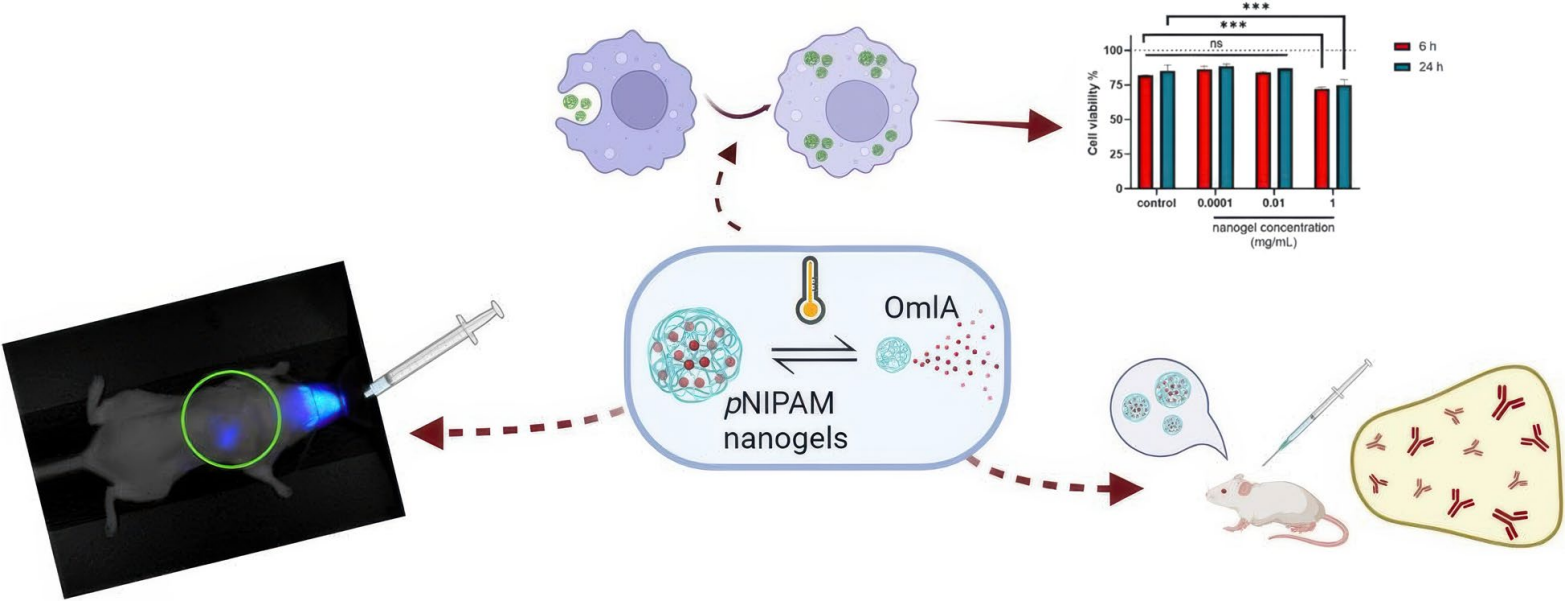
**Design of thermoresponsive nanogels based on pNIPAM and their performance in vitro, ex vivo, and in vivo using a mouse model**. Reprinted with permission from reference [2].

In response to these gaps, our research group has developed thermosensitive nanogels as vaccine carriers for the treatment of swine pleuropneumonia. In a first approach, Soriano et al. [2] reported the synthesis and physicochemical characterisation of thermoresponsive nanogels based on poly(N-isopropylacrylamide) (pNIPAM) and assessed their performance in vitro, ex vivo, and in vivo using a mouse model (Figure 3). The smart nanogels, approximately 250 nm in size with a transition temperature of 32 °C, were synthesised via precipitation polymerisation. Cytotoxicity assays conducted on various cell lines demonstrated high biocompatibility (> 70%). Efficient cellular internalisation was confirmed using confocal microscopy and flow cytometry.

The nanogels’ ability to protect and deliver antigens was analysed using outer membrane lipoprotein A (OmlA), a key virulence factor of *Actinobacillus pleuropneumoniae (*App*)*, the causative agent of porcine pleuropneumonia. The biodistribution of intranasally administered pNIPAM nanogels was tracked in vivo and ex vivo, revealing that the nanogels primarily remained in the lungs during the evaluation period. Additionally, the efficacy of the nanogel-based vaccine was assessed in vivo by measuring antibody titers in BALB/c mice inoculated with OmlA encapsulated in pNIPAM nanogels, compared to OmlA with aluminium hydroxide adjuvant. The results confirmed that the nanogels effectively stimulated a humoral immune response.

In their second study, Soriano et al. [3] investigated the use of these nanogels as carriers for the model antigen ovalbumin (OVA), with a focus on in vivo evaluations in both equine and murine models. The study included a thorough characterisation of the encapsulation efficiency and the physicochemical properties of OVA-loaded nanogels. In vitro biocompatibility assessments confirmed the favourable properties of these nanogels. In vivo evaluations in both equine and murine subjects assessed immunogenicity through antibody and splenic cell responses. The findings also suggest that nanogels may modulate immune responses by controlling antigen release kinetics. Results from in vitro assays indicated increased uptake of nanogels by APCs compared with free antigens.

### Nanogels as delivery systems

As previously discussed, nanogels are promising delivery systems due to their high encapsulation capacity, water solubility, and biocompatibility [10]. Among the various types of molecules that can be loaded into nanogels, antibiotics stand out due to the significant impact of bacterial infections on animal production. One example is clinical mastitis in dairy cattle, which has substantial adverse effects on animal health and welfare.

Addressing this condition requires effective treatment strategies that also support the judicious use of antibiotics. In this context, nanogel-based treatment offers potential advantages in enhancing precision and therapeutic efficacy.

#### Mastitis

Mastitis is one of the most significant challenges in veterinary medicine, particularly cow mastitis, due to the substantial economic losses it causes. Defined as inflammation of the mammary gland, mastitis can be triggered by a wide range of aetiological agents [61]. Among these, *Staphylococcus aureus* is particularly problematic due to its ability to survive intracellularly, which makes it difficult to control because high intracellular therapeutic drug concentrations are necessary [62].

**Figure 4:**
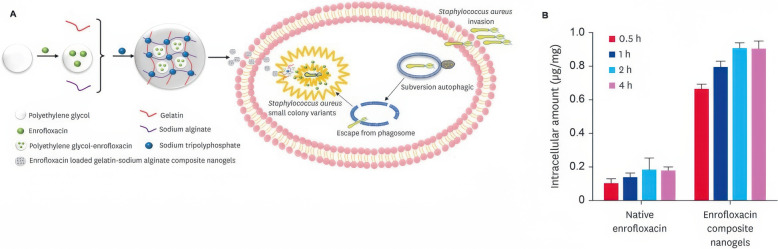
**Synthesis of sodium-gelatin composite nanogels and absorption of enrofloxacin into RAW 264.7 cells A) Schematic diagram describing the synthesis of enrofloxacin-loaded gelatin-sodium alginate composite nanogels**. **B** Uptake of native enrofloxacin and enrofloxacin composite nanogels in RAW 264.7 cells (*n* = 3) at different time points. Reproduced with permission from reference [65].

To address this issue, nanogel-based delivery systems have been developed as a safe and innovative strategy to overcome chronic infections and reinfections. For instance, Luo et al. [63] developed composite nanogels with loaded enrofloxacin to increase intracellular antibiotic concentration in the treatment of bovine mastitis caused by *S. aureus* (Figure 4). These nanogels were synthesised by electrostatic interaction using gelatin, sodium alginate, and CaCl_2_ as a cross-linker. The authors reported that the enrofloxacin-loaded composite nanogels improved the antibacterial activity and efficacy of antibiotics. Moreover, the composite nanogels could enhance the bioadhesion capacity of enrofloxacin to the *S. aureus* colony.

**Figure 5:**
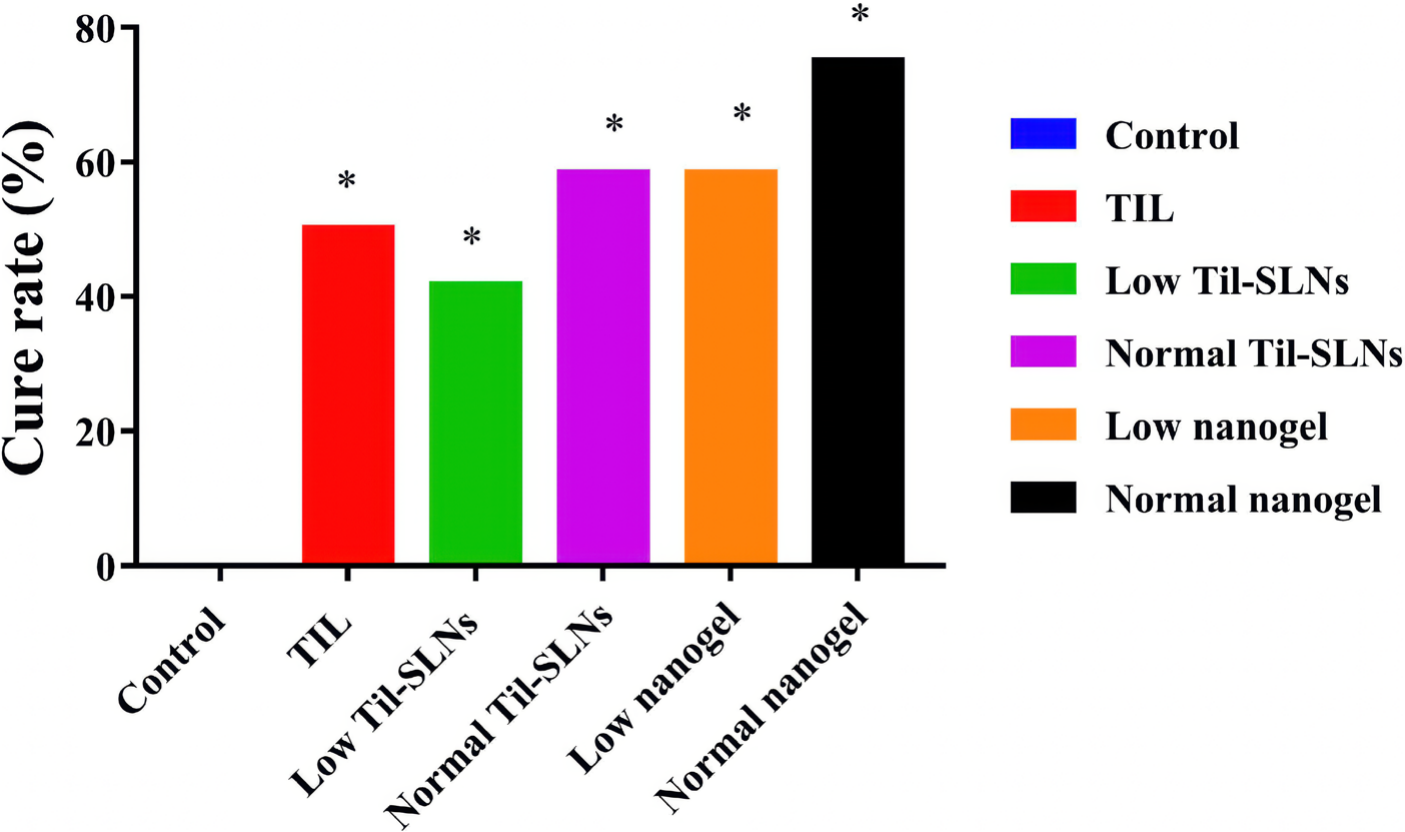
**The cure rate of various groups after administration**. *TIL* treatment with commercial tilmicosin injection (300 mg/gland per day), *Low TIL–SLNs* low dosage group of TIL–SLNs (150 mg/mammary per day), *Normal TIL–SLNs* normal dosage group of TIL–SLNs (300 mg/mammary per day), *Low nanogel* low dosage group of nanogel (150 mg/mammary per day), Normal nanogel: normal dosage group of nanogel (300 mg/mammary per day). *Statistically significant from control (*p* < 0.05) by chi-square test. Reproduced with permission from reference [66] (CC BY-NC 4.0).

In another attempt to reduce economic losses caused by *S. aureus*-induced mastitis, Zhou et al. [64] designed self-assembling sodium alginate–chitosan nanogels loaded with tilmicosin (TIL) as the antimicrobial agent. This formulation aimed to prolong intracellular retention of antibiotics by leveraging the combined antibacterial properties of chitosan and TIL, along with the wound-healing properties of sodium alginate (Figure 5). While in vitro results showed similar effects between TIL and TIL-loaded nanogels, the in vivo treatment outcomes were significantly improved with the nanogel formulation. The composite nanogels with tilmicosin exhibited higher in vivo antibacterial activity, even at half the usual TIL dose (150 vs 300 mg per mammary per day).

Mastitis often recurs and persists in cattle due to the ability of *S. aureu*s to form small colony variants (SCV) [65]. Florfenicol is generally used for this type of mastitis, but its effectiveness can be significantly reduced because the concentrations used are insufficient, and the antibiotic’s residence time in the mammary gland is not adequate for treatment [66]. To address this, Liu et al. [65] developed chitosan (CS) and sodium tripolyphosphate (TPP) nanogels through ionic gelation to encapsulate and deliver florfenicol specifically to SCV-infected sites, thus improving therapeutic concentrations and residence time. The researchers examined the release of the pH-sensitive antibiotic loaded into CS–TPP nanogels in a medium of pH 5.5 and 7.4. Results showed that nanogels loaded with florfenicol exhibited pH-triggered and sustained release over time. Additionally, the minimum inhibitory concentration (MIC) revealed that the dose of nanogels with florfenicol (0.25 µg/mL) improved susceptibility to the antibiotic compared to florfenicol solution (1 µg/mL). These findings indicated that the antibacterial activity was stronger in the nanogel due to the presence of CS and its antibacterial activity.

In vivo experiments in a mouse model of SCV-induced mastitis confirmed that florfenicol-loaded CS–TPP nanogels achieved higher healing rates than commercial florfenicol formulations. These results could be attributed to the presence of CS in the nanogel, the sustained release of the antibiotic over time leading to an increase in the healing rate, and the nanogels with florfenicol exhibiting stronger antimicrobial activity than the commercial antibiotic. The authors concluded that CS–TPP nanogels loaded with florfenicol provide a more effective therapeutic option against SCV-associated mastitis and represent a promising strategy for improving antibiotic therapy in veterinary medicine.

## One health

"One Health" is a transdisciplinary approach that recognises the interconnectedness of human, animal, and environmental health. This approach requires collaborative work across multiple disciplines, including human medicine, veterinary medicine, and environmental science. Globally, challenges such as antimicrobial resistance, environmental pollution, and the rise of chronic and multifactorial diseases are increasingly affecting human and animal health [67]. The global population of domestic and production animals increases each year, highlighting the need to develop effective and safe health products while minimising administration dose, pain, and adverse effects.

The antibiotic resistance era began in the mid-twentieth century with the widespread availability of penicillin. Since then, antibiotic resistance has substantially increased, fundamentally due to non-rational use of antibiotics. The unrestricted use of antibiotics in both human and animal production has exacerbated the spread of resistance. Frequently, antibiotics are employed as growth promoters at sub-lethal concentrations in animal production farms such as poultry, dairy, beef, goat, sheep, equine, pigs, and fisheries. These practices have driven the increase and spread of antimicrobial resistance from different families of antibiotics [68].

Reducing antibiotic dosage and developing alternative treatments are key research priorities in combating antimicrobial resistance [43]. In this context, nanotechnology plays a fundamental role in the development of novel solutions to combat antibiotic-resistant microorganisms. Nanoparticles can serve as vehicles for antibiotics, enhancing their delivery and efficacy, or act as antimicrobial agents themselves, providing innovative approaches to overcome resistance mechanisms [70].

## Conclusions and perspectives

In conclusion, the use of nanogels in veterinary medicine holds considerable promise for addressing challenges encountered in animal healthcare. This review highlights nanogels as a versatile platform with remarkable characteristics, including biocompatibility, tunable physicochemical properties, and the capacity for targeted drug delivery. These features enable precise control over the kinetics of therapeutic payload release, enhancing treatment efficacy while minimising adverse effects. The adaptable nature of nanogels allows for the encapsulation of diverse bioactive agents, ranging from small molecules to biomacromolecules, ensuring broad applicability across a spectrum of veterinary conditions.

Furthermore, the incorporation of nanogel-based formulations presents opportunities to overcome inherent limitations associated with conventional drug-delivery systems, such as poor bioavailability and rapid clearance. By harnessing nanotechnology, veterinary medicine may benefit from improved treatment outcomes, reduced dosing frequency, and increased patient compliance.

However, to realise the full clinical potential of nanogels in veterinary practice, further interdisciplinary research efforts are warranted to optimise formulation design, evaluate long-term safety profiles, and assess scalability for widespread adoption. Overall, the growing field of nanogel-based therapeutics represents a promising step forward in advancing the standards of care in veterinary medicine, paving the way for a new era of precision, efficacy and innovation in animal health management.

## Data Availability

Not applicable.
